# G Protein–Coupled Receptor Kinases: Crucial Regulators of Blood Pressure

**DOI:** 10.1161/JAHA.116.003519

**Published:** 2016-07-07

**Authors:** Jian Yang, Van Anthony M. Villar, Ines Armando, Pedro A. Jose, Chunyu Zeng

**Affiliations:** ^1^ Department of NutritionDaping HospitalThe Third Military Medical UniversityChongqingChina; ^2^ Department of CardiologyChongqing Key Laboratory for HypertensionChongqing Institute of CardiologyChongqing Cardiovascular Clinical Research CenterDaping HospitalThe Third Military Medical UniversityChongqingChina; ^3^ Division of Renal Diseases & HypertensionDepartment of MedicineThe George Washington University School of Medicine and Health SciencesWashingtonDC; ^4^ Department of Pharmacology and PhysiologyThe George Washington University School of Medicine and Health SciencesWashingtonDC

**Keywords:** blood pressure, G protein–coupled receptor kinases, G protein–coupled receptors, hypertension, High Blood Pressure, Hypertension

## Introduction

Hypertension, a complex trait determined by genetic, epigenetic, and environmental factors and their intricate interaction, is an important public health challenge worldwide because of its high prevalence and concomitant increase in the risk for cardiovascular disease. Unfortunately, the prevalence of hypertension is increasing in both developed and developing countries.^1^ As a consequence of the increase in global prevalence, the total number of adults with hypertension is predicted to increase to 1.56 billion in 2025.^2^ This prospect is daunting, given that, in 2010, high blood pressure was already the biggest single contributor to global mortality and disease burden.

The pathogenesis of essential hypertension is complex. Many organs and systems including kidneys, arteries, microcirculation, heart, immune system, nervous system, and endocrine factors are involved in the pathophysiology of hypertension. Among them, kidneys and arteries are major contributors to the development of hypertension.^3^, ^4^ Various agonists binding to plasma membrane receptors regulate renal sodium transport and fluid balance and maintain the equilibrium between vasoconstriction and vasodilation. Many of these agonists transmit their “information” via G protein–coupled receptors (GPCRs). GPCRs mediate cellular responses to diverse extracellular stimuli and play a vital role in the control of physiology and behavior.^5^ GPCR kinases (GRKs) interact with the agonist‐activated GPCRs to promote receptor phosphorylation and to initiate receptor desensitization.^6^ The wide variety of GPCRs that are responsible for optimal blood pressure control^7^ leaves no doubt that GRKs play a vital role in the regulation of blood pressure. A number of studies have shown that GRKs are associated with hypertension, blood pressure response to antihypertensive medicines, and adverse cardiovascular outcomes of antihypertensive treatment.^8^, ^9^, ^10^, ^11^ In this paper, we reviewed our evolving understanding of the role of GRKs in hypertension, summarized the current knowledge of GRK‐mediated regulatory mechanisms, and highlighted the potential for targeting GRKs in the treatment of hypertension. This information may advance our understanding of the role of GRKs in the control of blood pressure and provide novel insights into the field of translational medicine, especially regarding the design of new therapeutic approaches for the treatment of hypertension.

## Abnormal GPCR Function and Hypertension

**Figure 1:**
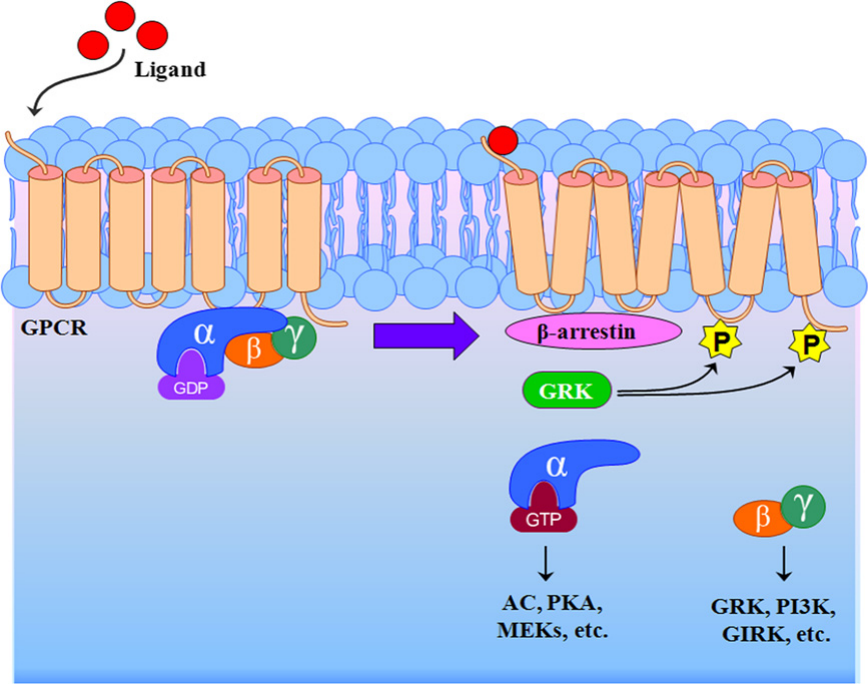
Schematic representation of the process of GPCR desensitization. On binding to their cognate ligands, GPCR activation initiates dissociation of cognate trimeric G protein, promoting GPCR phosphorylation by GRKs, leading to receptor association with members of the arrestin family, which inhibits further G protein activation. AC indicates adenylyl cyclase; GDP, Guanosine‐5′‐diphosphate; GIRK, G protein–gated inwardly rectifying potassium channel; GPCR, G protein–coupled receptor; GRK, G protein–coupled receptor kinase; GTP, Guanosine‐5′‐triphosphate; MEKs, mitogen‐activated protein/extracellular signal‐regulated protein kinase kinases; P, phosphorylation; PI3K, phosphatidylinositol‐3 kinase; PKA, protein kinase A.

GPCRs, the largest and most functionally diverse superfamily of cell‐surface receptors, share a common architecture consisting of 7 transmembrane domains connected by extracellular and intracellular loops.^5^ Upon stimulation, GPCRs interact with heterotrimeric G proteins that in turn dissociate into 2 functional units, namely, Gα and Gβγ subunits, both of which stimulate the activation of downstream proteins (Figure 1). In the vasculature, some GPCRs mediate vasoconstriction and/or vascular remodeling, such as angiotensin II (Ang II) type 1 receptor (AT_1_R), α‐adrenergic receptor (α‐AR), endothelin A receptor, and neuropeptide Y receptor, whereas other GPCRs induce vasodilatation and/or inhibition of vascular remodeling, including the acetylcholine receptor, β‐AR, the endothelin B receptor, and the dopamine receptor, among others. Similar to some renal tubular receptors (e.g., dopamine receptor, atrial natriuretic peptide receptor, AT_2_R, Mas receptor, and endothelin B receptor) decrease renal sodium reabsorption, whereas others including the AT_1_R, insulin receptor, and mineralocorticoid receptor increase renal sodium reabsorption. The balance between pro‐ and antihypertensive receptor activity is important to keep the blood pressure in the normal range. Abnormal GPCR functions lead to increased blood pressure; for example, increased AT_1_R function and impaired dopamine receptor function are found in hypertensive patients and hypertensive animal models.^12^, ^13^ The causes of abnormal GPCR function are complex and may include perturbation of DNA modification, receptor expression, and phosphorylation.^14^, ^15^ Among these modifications, GPCR phosphorylation is important. In hypertensive states, for example, dopamine D_1_ receptor (D_1_R) is hyperphosphorylated, which leads to uncoupling of the dopamine receptor from its G_αS_/effector protein complex and impairment of dopamine‐mediated natriuresis and vasodilation.^13^, ^15^, ^16^ It is known that the state of phosphorylation of GPCRs is modified by 2 kinds of enzymes. Kinases (e.g., GRKs) increase GPCR phosphorylation, whereas phosphatases (e.g., protein phosphatase 2A) bring about GPCR dephosphorylation. Renal protein phosphatase 2A activity is decreased in adult spontaneously hypertensive rats (SHRs) but increased in young (aged 2 weeks) SHRs, whereas GRK4 activity is markedly increased in hypertension.^16^, ^17^, ^18^ Nevertheless, the GRKs have received, by far, the most attention in abnormal GPCR phosphorylation in renal tubules in hypertension.

## Role of GRKs in the Regulation of Blood Pressure

### GRK Family Members

**Figure 2:**
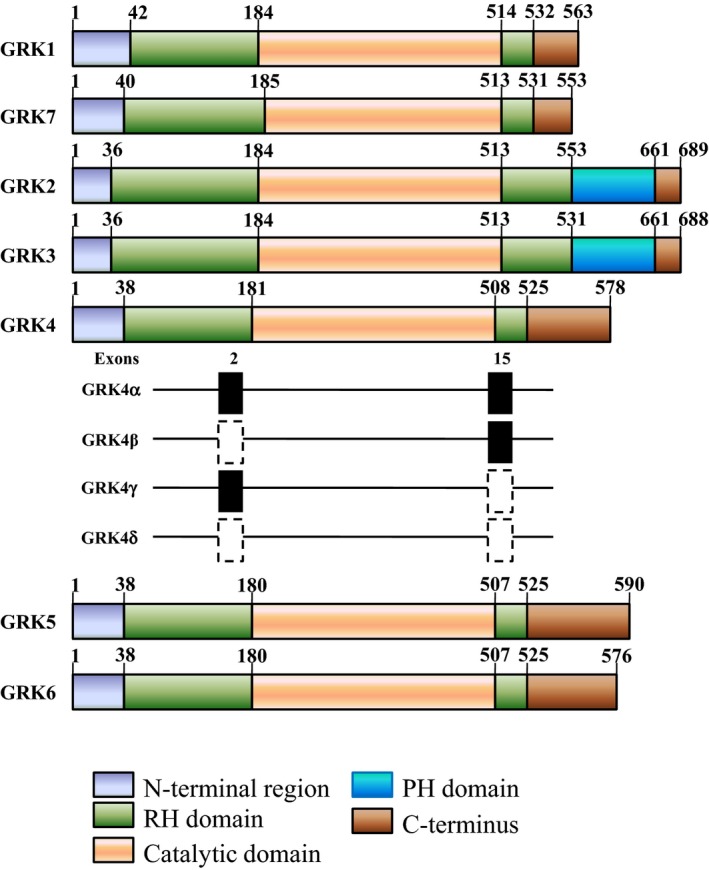
Structural domain distribution of GRKs. All GRKs possess an N‐terminal domain and a catalytic domain, both of which are followed by a Regulator of G protein signaling (RGS) homology domain, and a C‐terminal domain. GRK2 and GRK3 have another PH domain that interacts with G protein βγ subunits. The various isoforms of human GRK4 (GRK4α, GRK4β, GRK4γ, and GRK4δ) have differences in presence or in‐frame deletion of certain exons. The solid black square represents the presence of exon 2 and/or exon 15, whereas the dotted square represents the deletion of exon 2 and/or exon 15. GRK indicates G protein–coupled receptor kinase; PH, pleckstrin homology; RH, RGS homology.

Although there are >800 known GPCRs in the human genome, it is surprising that only 7 GRKs (GRK1–7) have been identified. The GRKs constitute a family of 7 serine/threonine protein kinases characterized by their ability to specifically recognize and phosphorylate agonist‐activated GPCRs. Based on divergent C‐terminal domain architecture and membrane‐targeting mechanisms, the GRKs are classified into 3 subfamilies: (1) the GRK1 subfamily, also known as the opsin kinase family, consisting of the rhodopsin kinase GRK1 and visual pigment kinase GRK7; (2) the GRK2‐like subfamily, also known as the β‐AR kinase family, consisting of GRK2 (β‐AR kinase 1) and GRK3 (β‐AR kinase 2); and (3) the GRK4‐like subfamily, consisting of GRK4, GRK5, and GRK6.^6^ All GRKs possess similar structural organization with an N‐terminal domain (≈185 amino acids), a catalytic domain (≈270 amino acids), and a C‐terminal domain (≈105–230 amino acids) (Figure 2). The carboxy tail region is GRK subtype‐specific; it is prenylated in the GRK1 subfamily, binds to Gβγ, contains a pleckstrin homology domain in the GRK2 subfamily, and has a C‐terminal helix/palmitoylation site in the GRK4 subfamily.^6^ The C‐terminal domain of GRKs is the most important determinant of subcellular localization and agonist‐dependent translocation.^19^ There is a nuclear localization sequence in all members of the GRK4 subfamily; the nuclear localization sequence in GRK5 and GRK6, but not GRK4, binds to DNA *in vitro*.^20^

The distribution of GRK subtype expression is different among subtypes. GRK1, GRK4, and GRK7 are expressed in limited numbers of tissues. GRK1 and GRK7 are found almost exclusively in the retina and regulate the opsins. GRK4 is expressed in testis, myometrium, kidney, artery, and intestine.^13^, ^15^, ^16^, ^17^, ^18^, ^21^ By contrast, other GRKs (GRK2, GRK3, GRK5, and GRK6) are expressed ubiquitously throughout the body. Accordingly, except for GRK1 and GRK7, other GRK members (GRK2–6) exert different physiological effects, specifically, the regulation of blood pressure by the cardiovascular system and the kidney.^8^, ^9^, ^10^, ^11^, ^16^, ^17^, ^18^, ^20^, ^21^, ^22^, ^23^, ^24^

#### G protein–coupled receptor kinase 2

The human *GRK2* (official name *ADRBK1*) gene locus maps at the long arm of chromosome 11: 11q13.2 by Ensembl, 11q13.1 by Entrez Gene (National Center for Biotechnology Information), and 11q13 by the HUGO Gene Nomenclature Committee (HGNC). Human *GRK2* cDNA encodes a protein of 689 amino acids (79.573 kDa) with an overall 98.0% amino acid and 92.5% nucleotide identity with bovine GRK2. The 23‐kb human *GRK2* gene consists of 21 exons interrupted by 20 introns, with a predicted transcription start site ≈246 bases upstream of the start ATG. The human *GRK2* gene has 12 highly conserved catalytic region subdomains in which 5 are encoded entirely within exons, specifically, exons 8, 9, 10, 11, and 12. Exons bounded by introns range in size from 52 (exon 7) to 163 bases (exon 18). The 2 largest exons represent the 5′‐flanking region (359 bases, including 113 bases of coding sequence) and the 3′‐coding plus noncoding region of the gene (>1200 bases). Sequence analysis of the 5′‐flanking/promoter region reveals many features characteristic of mammalian housekeeping genes, namely, the lack of a TATA box, an absent or nonstandardly positioned CAAT box, high GC content, and the presence of Sp1‐binding sites. The conserved region of the C‐terminal domain is important for enzyme–receptor interaction required for GRK2 to catalyze receptor phosphorylation.^25^ In addition, a Gβγ binding site of GRK2 is also localized in the C‐terminal pleckstrin homology domain. Phosphorylated Raf kinase inhibitory protein binds to the N terminus of GRK2, resulting in the inhibition of its function.^26^

##### Distribution of GRK2

GRK2 is ubiquitously expressed in mammals. In the cardiovascular system, GRK2 is expressed in the vascular endothelium, arterial smooth muscle, and myocardium.^27^, ^28^ GRK2 is also abundant in the kidney, especially in the renal proximal tubule.^29^ This renal expression indicates that GRK2 plays a vital role in the regulation of ion and fluid transport and, ultimately, blood pressure. GRK2 is expressed in both cytoplasm and the cell membrane. It shuttles between the cytosol and plasma membrane, anchoring to the latter through its pleckstrin homology and Gβγ binding domains at the C‐terminus.^30^ The subcellular localization of GRK2 at the mitochondrial outer membrane^27^, ^31^ may indicate a role of GRK2 in regulating mitochondrial biogenesis and cellular energy production. Indeed, GRK2 increases mitochondrial superoxide production and decreases oxygen consumption and ATP production.^32^

##### Regulation of GRK2 in the regulation of blood pressure

A role of GRK2 in the regulation of blood pressure has been shown in animal models with partial germline deletion, universal GRK2 knockdown, and targeted overexpression or knockdown in vascular smooth muscle and endothelial cells.^22^, ^33^, ^34^, ^35^, ^36^ GRK2 plays an important role in the regulation of blood pressure.^7^ Germline deletion of *Grk2* is lethal.^37^ GRK2 deficiency in global adult hemizygous mice (*Grk2* ^+/−^) has no effect on basal blood pressure but increases the vasodilator response to acetylcholine or isoproterenol and protects against Ang II–induced hypertension and vascular remodeling that is partially caused by increased nitric oxide bioavailability.^33^ Cohn et al also found in mice that inhibition of vascular smooth muscle GRK2 by either overexpression of the C‐terminal portion of GRK2 or vascular smooth muscle–specific ablation of GRK2 protein expression has no effect on blood pressure.^35^ This method of GRK2 silencing also had no effect on the elevated blood pressure resulting from unilateral renal artery stenosis.^35^ In contrast, overexpression of GRK2 in vascular smooth muscle in mice increases resting blood pressure.^34^ This study would agree with the report that GRK2 inhibits adiponectin function; adiponectin may be antihypertensive.^38^, ^39^ Portal hypertension caused by common bile duct ligation is also associated with an increase in GRK2 expression in the mesenteric artery.^40^ Consequently, deletion of GRK2 should result in a decrease in blood pressure; However, global knockdown of *Grk2* expression using small hairpin interfering RNA in male mice produces hypertension that is associated with vascular remodeling caused in part by increases in cell proliferation at age 6 months but not at 3 months.^22^ The causes of the differences are not known, but the differential effects of GRK2 on vasoconstriction and vasodilation may explain this apparently conflicting results. In small hairpin *Grk2* knockdown mice, for example, both phenylephrine‐induced contractile responses and isoproterenol‐mediated vasodilation are increased; which one dominates would eventually determine the physiological phenotype.^22^ After inhibition of GRK2 by either peptide inhibition or gene ablation, downregulation of GRK2 does not only increase β‐AR–mediated vasodilation but also enhances α_1D_‐AR–stimulated vasoconstriction and could explain the lack of effect on blood pressure of a decrease in GRK2 expression or function.^35^ Whether or not the discrepant results could be related to sex differences were not determined, but the small hairpin *Grk2* studies were performed only in male mice because the *Grk2* small hairpin RNA transgene was incorporated into the Y chromosome^22^; the sexes of the mice in the other studies were not given. Nevertheless, the transgenic overexpression of GRK5 in vascular smooth muscle increases blood pressure to a greater extent in male than in female mice.^23^ The discrepant results may also be related to the extent of downregulation of GRK2 in different tissues.

GRK2 is expressed to a greater extent than GRK5 in endothelial cells.^41^ Increased GRK2 expression in injured endothelial cells in injured liver leads to intrahepatic portal hypertension, and knockdown of *GRK2* in liver sinusoidal endothelial cells leads to an increase in portal pressure that is related to decreased endothelial nitric oxide synthase production of nitric oxide.^36^ Selective deletion of *Grk2* in the endothelium affects the aorta's receptor‐dependent and ‐independent vasoconstriction and increases vascular inflammation and tissue degeneration by increasing mitochondrial reactive oxygen species production, which is also associated with hypertension.^42^ Exercise decreases blood pressure, improves insulin sensitivity, and decreases mesenteric arteriolar and myocardial GRK2 expression in SHRs.^43^ The effects in mesenteric arterioles were prevented by mesenteric arteriolar overexpression of GRK2. In contrast, downregulation of endothelial vascular GRK2 expression in SHRs that is initiated at the prehypertensive stage (age 4 weeks) subsequently improves vascular insulin sensitivity that helps to limit the progression of hypertension.^43^ GRK2 impairs insulin sensitivity by binding to the insulin receptor substrate 1 but not to the insulin receptor.^44^ GRK2 expression in renal preglomerular vessels increases with aging in male but not in female rats.^45^ GRK2 regulates the blood pressure by modulating other receptor‐mediated vascular responses, including endothelin A receptor, neurotensin receptor 1, and P2Y receptor.^46^, ^47^, ^48^ The reasons why knockdown of GRK2 in mice in different studies leads to different effects on blood pressure need to be elucidated.

GRK2 also plays an important role in the renal regulation of sodium excretion and blood pressure. GRK2 keeps amiloride‐sensitive epithelial Na^+^ channels in the active state.^49^, ^50^, ^51^ GRK2 upregulates epithelial Na^+^ channel activity by a mechanism that depends not on its kinase activity but rather on the ability of the RGS homology domain of GRK2 to interact with and inhibit the α subunit of Gq/11, a negative regulator of epithelial Na^+^ channels.^50^ GRK2‐mediated phosphorylation of the C‐terminus of β–epithelial Na^+^ channels and phosphorylation of Nedd4‐2 prevent its ability to inhibit epithelial Na^+^ channel activity.^51^, ^52^ GRK2 negatively regulates neurotensin receptor 1 function; there are more neurotensin binding sites in the renal cortex than in the renal medulla, and they decrease sodium excretion, but the mechanism is not known.^47^ GRK2 also regulates the phosphorylation of renal D_1_R and D_1_R‐mediated natriuresis.^53^ In the human kidney, antisense oligonucleotides against *GRK2* and *GRK4* blunt the later stages of D_1_R desensitization; heparin, a nonselective GRK inhibitor, decreases GRK2 and GRK4 expression and attenuates the desensitization of D_1_R.^54^ Both GRK2 and GRK4 are involved in the desensitization of renal D_1_R in obese Zucker rats.^55^ Oxidative stress is involved in the regulation of GRK2 of D_1_R.^56^ Both *in vivo* and *in vitro* studies show that oxidative stress activates nuclear factor κB, causing an increase in protein kinase c (PKC) activity, which leads to GRK2 translocation and subsequent D_1_R serine hyperphosphorylation.^56^ The functional consequence of this phenomenon is the inability of D_1_R to inhibit Na^+^,K^+^‐ATPase activity and promote sodium excretion, which could contribute to the increase in blood pressure.^56^ Interestingly, we recently found that prenatal lipopolysaccharide exposure results in increased GRK2 expression, increased D_1_R phosphorylation, and impaired D_1_R‐mediated natriuresis and diuresis in the offspring. These findings suggest that a dysfunction of the renal D_1_R induced by abnormal GRK2 expression is also involved in fetal‐programmed hypertension.^57^ GRK2 is also involved in desensitization of D_2_R; D_2_R dysfunction is involved in the pathogenesis of hypertension.^58^, ^59^

##### Role of GRK2 in spontaneous hypertension

GRK2 expression in several tissues is increased in several diseases, including spontaneous hypertension in humans and experimental animals and in animal models of diabetes and insulin resistance.^28^ Gros et al reported that in SHRs, GRK2 expression is increased in both lymphocytes and aortic vascular smooth muscle cells and is accompanied by impairment of β‐adrenergic–mediated stimulation of adenylyl cyclase activity and β‐AR–mediated vasodilation.^60^ The impairment in β‐adrenergic–mediated aortic vasodilation and increased vascular GRK2 expression are observed in SHRs aged 10 and 15 weeks but not 5 weeks. Increased aortic vascular GRK2 expression is also present in the Dahl salt‐sensitive hypertensive rats after 4 weeks of a high salt diet.^60^ Oliver et al also reported impaired aortic β_1_‐ and β_2_‐AR–mediated vasodilation, but not β_3_‐AR–mediated vasodilatation, and increased aortic expression of GRK2 in adult SHRs^61^; however, this group did not find such differences in the mesenteric artery of adult Wistar‐Kyoto (WKY) rats and SHRs.^61^ Moreover, in rats made hypertensive by L‐NAME, their aortas had increased β_2_‐AR–mediated vasodilation and decreased GRK2 expression; their mesenteric arteries had decreased β_2_‐AR‐mediated vasodilation, without changes in GRK2 expression—opposite to that found in SHRs.^61^ Whether or not inconsistencies are present in other models of hypertension remain to be determined.

GRK2 is expressed in peripheral blood mononuclear cells and lymphocytes.^7^, ^62^ GRK activity and GRK2 expression are increased in lymphocytes of hypertensive humans and experimental models of hypertension.^60^, ^63^, ^64^ Lymphocyte *GRK2* mRNA expression directly correlates with systolic blood pressure and plasma norepinephrine levels.^64^ GRK2 in lymphocytes is elevated >30% among persons with systolic blood pressure >130 mm Hg. GRK2 protein expression in lymphocytes is also increased about 2‐fold, and its activity increased >40% in African Americans, a population at higher risk for hypertension and cardiovascular complications compared with other groups.^64^

#### G protein–coupled receptor kinase 3

The human *GRK3* (official name *ADRBK2*) gene locus maps at the long arm of chromosome 22: 22q12.1 by Ensembl and Entrez Gene and 22q11 by HGNC. Similar to GRK2, the *GRK3* gene also has 21 exons ranging in size from 52 to 163 bases. The amino acid sequence of human *GRK3* is 84%, identical to that of human *GRK2*. Similarly, bovine GRK3 has 85% amino acid identity with GRK2. The most highly conserved region between GRK3 and GRK2 is the protein kinase catalytic domain, which has only 12 amino acid differences (95.0% identity), 4 of which are conservative substitutions (96.7% conservative). In contrast, the amino‐terminal domain (80.7% identity, 89.8% conservative) and carboxyl‐terminal domain (76.6% identity, 88.9% conservative) are less well conserved.

GRK3 belongs to the GRK2 subfamily and is ubiquitously expressed in the body; however, unlike GRK2 and GRK5, GRK3 is not expressed in endothelial cells.^41^ In contrast, in cardiac myocytes, the GRK2 subfamily expression is GRK5 to GRK3 to GRK2. Many studies have focused on the role of GRK2 and GRK3 on cardiac function.^8^, ^65^ GRK3 and GRK2, however, have distinct roles in receptor selectivity in cardiac myocytes and receptor‐mediated regulation of cardiac function; GRK3 has selectivity for the α_1B_‐ARs and for the thrombin receptor but exhibits less efficacy at β_1_‐ARs than GRK2.^65^ Their subcellular distribution in cardiac myocytes is also different. Consequently, GRK2 expression is increased in intercalated discs in rats with spontaneously hypertensive heart failure, whereas GRK3 expression is increased in cross‐striations in α‐actinin and Gα at Z‐lines.^66^

Unlike the prohypertensive action of GRK2,^33^, ^34^ GRK3 may play a protective role in the regulation of blood pressure. Cardiac myocytes of spontaneously hypertensive heart failure rats have increased expression of GRK3 and GRK6 and altered distribution, including that of GRK2.^66^ GRK3 expression in human lymphocytes significantly and inversely correlates with systolic and diastolic ambulatory blood pressure.^9^ The protective role for GRK3 in the regulation of blood pressure is supported by findings in transgenic mice in which cardiac myocyte–restricted inhibition of endogenous GRK3 causes hypertension because of increased cardiac output caused in part by cardiac myocyte α_1_‐AR hyperresponsiveness.^67^ GRK3 is important in α_1B_‐AR signaling; GRK5 has a partial effect, whereas GRK2 has no effect.^68^ Although α‐ARs are key regulators of vascular resistance and GRK3 is expressed in the vasculature, it remains unknown whether or not GRK3 can regulate the blood pressure by exerting some functions in vascular resistance. In addition, GRK3 reportedly regulates the phosphorylation of D_1_R^53^ and D_2_R; however, its physiological consequence is not clear.

#### G protein–coupled receptor kinase 4

The human *GRK4* gene locus (4p16.3) is embedded in a gene cluster region on chromosome 4p16 that includes genes encoding dopamine receptor type 5 (4.p16.1) and α‐adducin (4p16.3), 2 variants of which (*ADD1* and *GRK4*) are linked to hypertension.^16^, ^69^, ^70^ The human *GRK4* gene is composed of 16 exons extending over 75 kb of DNA. Alternative splicing generates 4 isoforms of human GRK4 mRNA that differ in the presence or absence of exon 2 at the N‐terminal region and exon 15 in the C‐terminal region: GRK4α (578 amino acids, 66.5 kDa) is the full‐length isoform; GRK4β (546 amino acids, 62.9 kDa) lacks only the N‐terminal exon 2 (32‐codon deletion); GRK4γ (532 amino acids, 61.2 kDa) lacks only the C‐terminal exon 15 (46‐codon deletion); and the shortest splice variant is GRK4δ (500 amino acids, 57.6 kDa), missing both exons 2 and 15.^71^ In addition, 5 GRK4 splice variants (GRK4A–E) in rat and only 1 GRK4 splice in mouse have been reported. Only the GRKα isoform in humans, GRK4A in rats, and only GRK4 reported in mice are closely homologous (≈70%), whereas the mouse and rat GRK4 sequences retain 90% identity.

##### Distribution of GRK4

As noted previously, GRK2, GRK3, GRK5, and GRK6 are ubiquitously expressed, whereas GRK4 is expressed in a limited number of tissues. GRK4, for example, is abundantly expressed in the testes and human myometrium and, to a lesser extent, in a few other tissues, including the artery, brain, kidney, and intestine, but has minimal expression in the normal heart.^16^, ^71^, ^72^ The distinct distribution of GRK4 indicates its vital role in the regulation of blood pressure. In both WKY rats and SHRs, GRK4 expression is strongly expressed in subapical membranes of renal proximal tubules (S1 and S3 segments), thick ascending limbs of the loop of Henle, and the distal convoluted tubules and much less in glomeruli.^16^, ^72^, ^73^ GRK4 is also present in rat renal resistance vessels, but its physiological function remains unclear. Basal GRK4 expression in the renal cortex is much higher in SHRs than in WKY rats, whereas cardiac GRK4 expression is similar in the 2 rat strains, indicating that the increased GRK4 expression in hypertension has organ specificity.^72^

In our recent studies, we found that GRK4 is also expressed in the tunica media and adventitia of arteries from Sprague‐Dawley rats and C57BL/6J mice.^21^ GRK4 is expressed in both large and small vessels, including the thoracic aorta, superior mesenteric artery, carotid arteries, and renal artery, and there is no difference in GRK4 expression in these vessels. The physiological significance of GRK4 at the tunica adventitia, however, remains to be determined because GRK4 in this layer does not participate in the Ang II–mediated vasoconstriction.^21^ In addition, we found that GRK4 is expressed in the myocardium, which is involved in the regulation of myocardial ischemia. Overexpression of GRK4 or its variants in mice contributes to the aggravation of the ischemia induced by myocardial injury (L.P. Li, J. Yang, and C.Y. Zeng Ph.D., unpublished data, 2016).

##### Regulation by GRK4 of blood pressure

The dopaminergic system and the renin–angiotensin system are important regulators of sodium balance and blood pressure, which are relevant to the pathogenesis and/or maintenance of hypertension.^13^, ^15^, ^16^, ^17^, ^18^, ^54^, ^55^, ^56^, ^57^, ^59^, ^71^, ^72^, ^73^, ^74^, ^75^, ^76^, ^77^, ^78^ The dopaminergic system exerts a paracrine regulatory role on renal sodium transport in the proximal tubule via its 5 receptor subtypes. Dopamine receptors, pharmacologically grouped into D_1_‐like (D_1_ and D_5_) and D_2_‐like (D_2_, D_3_, and D_4_) receptors, as with the Ang II receptors (AT_1_R and AT_2_R), are expressed in brush border and basolateral membranes of renal proximal tubules. AT_1_R mediates the vast majority of renal actions of Ang II, including renal tubule sodium reabsorption. In contrast to the stimulatory effect of Ang II on sodium transport in renal proximal tubules, the major consequence of the activation of dopamine receptors is the inhibition of sodium transport.^13^, ^15^, ^16^, ^17^, ^18^, ^54^, ^55^, ^56^, ^57^, ^59^, ^71^, ^72^, ^73^, ^74^, ^75^, ^76^, ^77^, ^78^ Increasingly, studies show that GRK4 plays an important physiological role in the long‐term control of blood pressure and in sodium homoeostasis via the regulation of the renal D_1_R, D_3_R, and AT_1_R.

Studies have shown that increased GRK4 activity causes impaired renal D_1_R function in hypertension. GRK4 activity is increased in the kidneys of humans with essential hypertension, but the increased activity is caused not by increased renal GRK4 protein expression but rather by constitutively active variants of GRK4.^16^ In human renal proximal tubule cells, GRK4 constitutively phosphorylates the D_1_R in the absence of agonist activation; however, inhibition of GRK4 activity or depletion of GRK4 blunts the D_1_R desensitization.^54^ The abundance of basal GRK4 and serine‐phosphorylated D_1_R in renal cortical membranes are much higher in SHRs relative to WKY rats.^72^ Selective renal cortical inhibition of GRK4 expression decreases serine‐phosphorylated D_1_R to a greater extent in SHRs than in WKY rats; it also increases sodium excretion and attenuates the increase in arterial blood pressure with age in SHRs but not in WKY rats.^72^ Similar to GRK2, the dysfunction of the renal D_1_R induced by abnormal GRK4 expression is also involved in fetal‐programmed hypertension.^57^ In renal proximal tubules, however, GRK4 is more important than other GRKs in the desensitization of D_1_R and D_3_R.^54^, ^73^ These findings suggest the crucial role of renal GRK4 in the D_1_R‐ and D_3_R‐mediated control of sodium excretion and blood pressure.

The 3 human *GRK4*γ single nucleotide polymorphisms (SNPs; 65R>L, 142A>V, and 486A>V) markedly impair D_1_R‐mediated cAMP accumulation in the kidney, which is not due to differences in the quantity of the expression of either D_1_R or GRK4.^16^ Compared with *GRK4*γ wild‐type transgenic mice, *GRK4*γ142V transgenic mice are hypertensive and fail to increase urine flow and sodium excretion in response to the D_1_R agonist fenoldopam; the decreased ability of fenoldopam to inhibit renal sodium transport is also observed *in vitro*.^16^ The increase in blood pressure in *GRK4*γ142V transgenic mice is not related to chromosomal integration, copy number, or renal human GRK4 mRNA level but rather is mainly caused by the effect of the *GRK4*γ142V transgene acting via D_1_R.^76^ *In vitro* studies showed that in single‐variant (142A>V, 65R>L, or 486A>V) or double‐variant (65L/486V) *GRK4*γ‐transfected Chinese hamster ovary cells, there is an increase in basal D_1_R phosphorylation and impairment of D_1_R‐mediated cAMP production.^16^ We also found that the function of D_3_R is also impaired in the *GRK4*γ*1*42V‐transfected human renal proximal tubule cells (J. Yang, MD, PhD, et al, unpublished data, 2016).

In addition to GRK4 regulation of D_1_R and D_3_R in the renal proximal tubule, there is also evidence that GRK4 regulates AT_1_R expression and activity in this nephron segment. The *GRK4* gene variants that are associated with hypertension increase renal proximal tubule AT_1_R expression and activity. In *GRK4*γ142V transgenic mice, due to the inhibition of renal histone deacetylase type 1 (but not histone deacetylase type 2) activity, renal AT_1_R expression and activity are increased, which leads to increased blood pressure. In contrast, AT_1_R blockade or deletion of the *AT_1_R* gene normalizes the hypertension in *GRK4*γ142V transgenic mice.^77^ Our recent study also showed that, due to higher nuclear factor κB activity with more nuclear factor κB bound to the AT_1_R promoter, both AT_1_R expression and AT_1_R‐mediated vasoconstriction are higher in the aorta of *GRK4*γ142V than *GRK4*γ wild‐type transgenic mice.^21^ In *GRK4*γ142V transgenic mice. Ang II causes a greater increase in systolic blood pressure, whereas infusion of the AT_1_R antagonist candesartan causes a greater decrease in blood pressure in *GRK4*γ142V transgenic mice than their wild‐type counterparts.^21^, ^77^ Similarly, renal AT_1_R expression is also enhanced in *GRK4*γ486V transgenic mice fed a high salt diet, which may contribute to the salt‐sensitive phenotype of these mice; however, the *GRK4*γ wild‐type transgene prevents salt‐sensitive hypertension.^77^, ^78^

##### Role of GRK4 in human essential hypertension

The *GRK4* locus on human chromosome 4p16.3 is linked to essential hypertension and salt sensitivity.^79^, ^80^, ^81^, ^82^, ^83^, ^84^, ^85^ Three missense SNPs ( ie, 65R>L, 142A>V, and 486A>V) in the coding region of *GRK4*γ are associated with increased blood pressure. Depending on the genetic background of the mouse, mice overexpressing the *GRK4*γ wild‐type transgene are normotensive and salt‐resistant, whereas *GRK4*γ142V transgenic mice have high blood pressure even with normal NaCl intake.^16^, ^76^, ^77^ In contrast, *GRK4*γ486V or *GRK4*γ65L transgenic mice become hypertensive only after an increase in sodium intake.^77^, ^78^

A number of studies have shown the genetic association of the 3 *GRK4* SNPs with human essential hypertension in several ethnic groups. The association between *GRK4*486V and essential hypertension was found in Italian and Euro‐Australian populations.^79^, ^80^ In a study of northern Han Chinese participants, the *GRK4*65L, *GRK4*142V, and *GRK4*A486 haplotypes are associated with a 6‐fold higher risk of systolic and diastolic hypertension.^81^ In these same Han Chinese participants, *GRK4*486V alone is associated with hypertension.^82^ In an African‐derived semi‐isolated Brazilian population, the combination of *NOS3* rs1799983 and *GRK4*486V is associated with hypertension.^83^ *GRK4*486V is also associated with salt sensitivity in a Euro‐American population.^84^ In a Japanese cohort, the presence of all 3 *GRK4* variants impaired the natriuretic effect of a dopaminergic agonist and correctly predicted the presence of salt‐sensitive hypertension in 94% of cases.^85^ The single‐locus model with only *GRK4*142V is 78.4% predictive, whereas a 2‐locus model of *GRK4*142V and aldosterone synthase CYP11B2 is 77.8% predictive of low‐renin hypertension.^85^ Some reports, however, do not show the association between *GRK4* variants and hypertension that may be related to the failure to study all of the *GRK4* variants or to the age of the participants.^86^, ^87^

#### G protein–coupled receptor kinase 5

The human *GRK5* gene locus maps to the long arm of chromosome 10: 10q26.11 by Ensembl, Entrez Gene, and HGNC. GRK5, a 590‐amino acid protein kinase, has 34.8% and 47.2% amino acid identities with GRK2 and GRK1, respectively. *GRK5* contains a centrally located protein kinase catalytic domain of 238 amino acid residues flanked by N‐terminal and C‐terminal regions of 193 and 159 amino acid residues, respectively. The atomic structure of GRK5 has been shown to be aligned in manner different from the other GRKs.^88^ *GRK5* mRNA is found most abundantly in the lung, heart, retina, and lingual epithelium but is minimally expressed in brain, liver, kidney, and testis. Many studies of GRK5 have focused on its role in the exacerbation of pathological cardiac hypertrophy^89^; however, GRK5 is also involved in the pathogenesis of hypertension. GRK5, as with GRK2, for example, is also increased in lymphocytes from hypertensive humans and animal models of hypertension.^90^

The intracardiac injection of adenovirus encoding the amino‐terminal region of GRK5 increases the already elevated blood pressure of SHRs.^91^ GRK5 overexpression in vascular smooth muscle cells in mice increases blood pressure. The hypertension in male GRK5 transgenic mice is caused in part by a decrease in β_1_‐AR activity, whereas high blood pressure in female mice is caused by an increase in activity of AT_1_R.^23^ *Grk5* knockout mice have impaired glucose tolerance and insulin sensitivity, indicating that GRK5 is a positive regulator of insulin sensitivity.^92^ Increased expression of GRK5 is associated with different animal models of hypertension, including Ang II‐, norepinephrine‐, and L‐NAME–induced hypertension.^93^, ^94^ There is a nuclear redistribution of GRK5 in hypertensive heart‐failure‐prone rats.^95^ The physical association of AT_1_R and GRK5 is increased in the heart in congestive heart failure but is reversed by exercise training.^96^ *In vitro* studies showed that both AT_1_R and D_1_R can be the substrates for GRK5. The agonist‐dependent phosphorylation of the AT_1_R is substantially increased in human embryonic kidney cells overexpressing GRK5, GRK2, or GRK3.^97^ GRK4 and GRK5 impair both the sensitivity and maximum response of D_1_R, whereas GRK2 and GRK3 impair only the sensitivity of D_1_R to agonist stimulation.^16^, ^98^ Whereas HDAC1 is involved in the increase in *GRK4*γ142A>V‐mediated increase in renal AT_1_R expression,^77^ HDAC5 is associated with the GRK5‐regulated gene transcription in heart failure.^99^

*GRK5*Leu41 is a nonsynonymous polymorphism of *GRK5*, common in African‐Americans, in which leucine is substituted for glutamine at position 41. A study showed that the *GRK5*Leu41 polymorphism decreased the risk for adverse cardiovascular response but not for the blood pressure response to antihypertensive medication.^100^ Another study showed a pharmacogenomic interaction between *GRK5*Leu41 and β‐AR‐blocker treatment in which the presence of the *GRK5*Leu41 polymorphism was associated with decreased mortality in African‐Americans with heart failure or cardiac ischemia.^101^

#### G protein–coupled receptor kinase 6

The human *GRK6* gene locus maps to the long arm of chromosome 5: 5q35.3 by Ensembl, 5q35 by Entrez Gene, and 5q35 by HGNC. The crystal structure of GRK6 has been deciphered.^102^ GRK6 has higher homology with GRK5 (70.1% amino acid identity) compared with GRK2 (37.4%) and GRK1 (47.1%).The structure of GRK6 reveals a putative phospholipid binding site near the N‐terminus and structural elements within the kinase substrate channel that influence GPCR access and specificity.^102^ GRK6 is expressed ubiquitously throughout the body, including the brain, skeletal muscle, pancreas, and myometrium and at lower levels in the heart, lung, kidney, placenta, and liver.

As with the other GRKs, GRK6 also regulates the β‐AR and AT_1_R.^103^, ^104^, ^105^ GRK6 but not GRK2 or GRK5 is involved in the desensitization of calcitonin gene‐related peptide.^106^ GRK6 also regulates Na^+^/H^+^ exchanger regulatory factor^107^; Na^+^/H^+^ exchanger regulatory factor and Na^+^/H^+^ exchanger type 3 are involved in the regulation of renal sodium transport. Although D_1_‐like receptor‐mediated inhibition of renal sodium Na^+^/K^+^/ATPase activity requires Na^+^/H^+^ exchanger regulatory factor 1,^108^ and dopamine receptors are important in the regulation of renal sodium transport and blood pressure,^59^, ^74^ Na^+^/H^+^ exchanger regulatory factor 1 *per se* does not regulate blood pressure.^109^ Both D_1_‐ and D_2_‐like dopamine receptors are physiological targets of GRK6. Inhibition of GRK6 prevents agonist‐induced desensitization of intestinal D_1_‐like receptors in rat intestinal epithelial cells.^107^ Dopamine D_2_R hypersensitivity occurs with disruption of the *GRK6* gene in mice.^110^ Consequently, abnormalities of the *GRK6* gene can lead to D_2_R supersensitivity, which can result in dysfunction of D_2_R in the regulation of natriuresis and blood pressure.

The expression of GRK6 is affected by hypertensive status and associated with hypertension‐induced complications. Renal GRK6 levels are lower in hypertensive participants and SHRs than their normotensive controls^111^; however, GRK6 expression is increased in spontaneously hypertensive heart failure rats.^66^ Moreover, subcellular redistribution of GRK6 in spontaneously hypertensive heart failure rats is also involved in abnormal remodeling of cardiac myocytes in hypertensive hypertrophy and failure.^66^ GRK6 is also important in the negative regulation of inflammation,^112^ which is intimately involved in the regulation of blood pressure and development of hypertension.

## GRK Gene Variants and Their Roles in Hypertension

Since the discovery of a linkage between GRKs and cardiovascular disease including hypertension and heart failure, GRKs—especially GRK2 and GRK4—have been considered pharmaceutical targets for the treatment of cardiovascular disease. Moreover, GRK gene variants are also important for guiding therapeutic antihypertensive strategies.^100^, ^113^

Current evidence shows that common variants of GRK4 are associated with human essential hypertension and predict the blood pressure response to antihypertensive medicines. Our recent study in hypertensive Japanese participants showed that carriers of *GRK4*142V had a greater decrease in systolic blood pressure in response to angiotensin receptor blockers than noncarrier hypertensive patients. In contrast, those with variants only at *GRK4*486V were less likely to achieve the blood pressure goal in response to angiotensin receptor blockers than those with no variants.^10^ Nevertheless, in a small cohort of Japanese hypertensive participants, those with *GRK4*486V has a good antihypertensive response to a low‐salt diet or diuretics.^114^ The association between GRK4 variants and the response to antihypertensive treatment has also been confirmed in American and European hypertensive participants. Results from the African American Study of Kidney Disease and Hypertension Study suggest a sex‐specific relationship between *GRK4*A142V and blood pressure response among African‐American men with early hypertensive nephrosclerosis. Men with *GRK4*A142 were less responsive to metoprolol if they also had a *GRK4*L65 variant, but the additive effect of A142 and L65 variants on blood pressure was not found in women.^115^ Another study from the Pharmacogenomic Evaluation of Antihypertensive Responses trial involving hypertensive African and Euro‐American participants found that *GRK4*65L and *GRK4*142V variant alleles and increasing copies of the variant *GRK4*65L and *GRK4*142V haplotypes are associated with reduced response to β‐blocker monotherapy. Moreover, all 3 *GRK4* variants (65L, 142V, and 486V) are associated with increased risk for the primary outcome (first occurrence of all‐cause death, nonfatal myocardial infarction, or nonfatal stroke) in pooled white and Hispanic participants.^11^ European hypertensive patients who are homozygous for *GRK4*65L and *GRK4*142V have been reported to need more antihypertensive treatment, especially diuretic therapy, to reach the same mean arterial blood pressure as homozygous carriers of only 1 variant or heterozygous/wild‐type carriers of R65L, A142V, and A486V alleles.^116^ These results suggest that the presence or absence of *GRK4* gene variants may be important determinants in guiding therapeutic antihypertensive strategies.

GRK2 may also influence to response to some antihypertensive medicines. The SNPs in the *GRK2* gene are more common in African‐Americans, who have a higher risk for increased blood pressure.^100^ Specifically, 2 *GRK2* SNPs are associated with blood pressure response to antihypertensive medicines. Compared with African‐American patients with the rs4930416 homozygote, those with a heterozygote (rs4930416, A>C) have similar blood pressure at baseline but greater blood pressure reduction with hydrochlorothiazide. Diastolic blood pressure, but not systolic blood pressure, response to atenolol also differs by rs4930416 genotype.^100^ There are also trends toward different diastolic blood pressure and systolic blood pressure responses with another *GRK2* gene SNP, rs1894111, in Euro‐American patients receiving hydrochlorothiazide; however, this SNP is neither associated with altered blood pressure response to atenolol nor response in African American patients.^100^

## Conclusions

Overwhelming data demonstrate that GRKs (GRK2, GRK3, GRK4, GRK5, GRK6), via different mechanisms, play important roles in the regulation of blood pressure (Table).^7^, ^8^, ^18^, ^59^, ^71^, ^74^, ^90^, ^113^, ^117^ Aberrant GRKs in the cardiovascular system and kidney are involved in the pathogenesis of hypertension. Modulation of GRK activity has yielded promising results in the regulation of blood pressure, alleviating cardiovascular and renal dysfunction in a wide variety of animal models and cell culture systems. Genetic studies have found a strong association between GRK gene variants and hypertension. Identification of GRK variants is important in choosing antihypertensive medication and represents a valuable pharmaceutical target for novel therapeutic approaches in the treatment of hypertension. Increased understanding of GRKs in the regulation of blood pressure may give us a novel concept for the pathogenesis of hypertension and provide new therapeutic antihypertensive strategies in the future.

**Table 1:** Summary of GRK Family and Hypertension

GRK Isoform	Tissue Distribution	GRK Modification	Effects of GRK Modification on Blood Pressure and Related GPCRs	GRK Expression and Activity in Hypertension
GRK2	Ubiquitous expression	VSM‐targeted overexpression	Impairs β‐AR induced vasodilation^34^; increases resting blood pressure^34^	Increased GRK2 expression and GRK activity in lymphocytes and arteries in hypertensive patients and SHRs^60^, ^63^, ^64^; increased GRK2 expression in conductance and resistance vessels in SHRs^61^, ^117^; decreased GRK2 expression in conductance vessels and no change in GRK2 expression in resistance vessels in L‐NAME‐ induced hypertensive rats^61^; increased GRK2 expression and GRK activity in mesenteric artery of sedentary SHRs^43^; increased renal GRK2 expression in obese rats^29^; increased renal GRK2 expression in offspring of lipopolysaccharide‐treated dams^57^
		Hemizygous mice (GRK2^+/−^)	No effect on baseline blood pressure but protects against Ang II–induced hypertension and vascular remodeling^33^	
		Global knockdown using a shRNA	Results in spontaneous hypertension^22^; increases both vasoconstriction in response to PE and vasodilatation in response to β‐AR stimulation^22^	
		VSMC‐specific ablation of GRK2	No effect on baseline blood pressure^35^; increases β‐AR–mediated vasodilation, but also enhances α_1D_AR‐stimulated vasoconstriction^35^	
		Selective deletion of endothelial GRK2	Blood pressure not measured; blunts vasoconstriction to different agonists^42^	
		Gene depletion Renal proximal tubule GRK2 gene depletion	Blunts desensitization of arterial ETAR^46^; promotes insulin‐induced vasodilation of mesenteric arteries in SHRs^43^blunts desensitization of renal proximal tubule D_1_R^54^	
GRK3	Ubiquitous expression	Cardiac‐restricted GRK3 inhibition	Increases blood pressure and cardiac output^67^; increased cardiac myocyte α_1_‐AR responsiveness^67^; attenuates cardiac dysfunction caused by pressure overload^24^	No significant difference in GRK3 expression in the lymphocytes of hypertensive and normotensive patients^9^
GRK4	Testes, myometrium, brain, intestines, kidney, and artery	Overexpression of human *GRK4*142V	Increases blood pressure (normal salt diet)^16^, ^77^; impairs renal D_1_R function^16^; increases AT_1_R expression in the kidney and artery^21^, ^77^; increases in systolic blood pressure response to Ang II^21^	Increased renal GRK4 expression in SHRs^72^; increased renal GRK activity in hypertensive subjects^16^; increased renal GRK4 expression and normalized by rosiglitazone (insulin sensitizer) in obese Zucker rats^55^; increased renal GRK4 expression in offspring of lipopolysaccharide‐treated dams^57^
		Overexpression of human *GRK4*486V	Increases blood pressure (on high salt diet) and renal AT_1_R expression^78^; increases in basal D_1_R phosphorylation and impairs the function of D_1_R^16^	
		Overexpression of human *GRK4*65L	Increase basal D_1_R phosphorylation and impairs D_1_R‐mediated cAMP production^16^	
		*GRK4* gene depletion	Increases sodium excretion, attenuates the increased blood pressure and renal serine‐phosphorylated D_1_R in SHRs^72^; blunts the D_1_R desensitization in human RPTCs^54^; blocks D_1_R phosphorylation and restores D_1_R‐ mediated cAMP accumulation in RPTCs from hypertensive participants^16^	
GRK5	Ubiquitous expression	VSM‐specific overexpression	Increases blood pressure^23^	Increased GRK5 expression in Ang II–treated VSMCs^93^; increased GRK5 expression in aortas of Ang II– and norepinephrine‐induced hypertension^93^; no significant difference in GRK5 expression in the lymphocytes of hypertensive and normotensive patients^9^
		Global knockout	Increases insulin resistance^92^	
		Overexpression of GRK5	Increases agonist‐dependent phosphorylation of the AT_1_R or D_1_R^97^, ^98^	
GRK6	Ubiquitous expression	Global knockout	Causes striatum D_2_R supersensitivit^110^	Decreased renal GRK6 expression in hypertensive participants and SHRs^111^; increased myocardial GRK6 expression in SHHF rats^66^
		Inhibition using antibody	Prevents intestinal D_1_R desensitization^107^	

Ang II indicates angiotensin II; AR, adrenergic receptor; AT_1_R, angiotensin II type 1 receptor; D_1_R, dopamine D_1_ receptor; ETAR, endothelin A receptor; GPCR, G protein–coupled receptor; GRK, G protein–coupled receptor kinase; PE, phenylephrine; RPTC, renal proximal tubule cell; SHHF, spontaneously hypertensive heart failure; SHR, spontaneously hypertensive rat; shRNA, small hairpin RNA; VSM, vascular smooth muscle; VSMC, vascular smooth muscle cell.

## Sources of Funding

These studies were supported in part by grants from National International Technology special grant (2014DFA31070), National Natural Science Foundation of China (81570379, 81100500), grants from National Institutes of Health, United States (R37HL023081, R01DK039308, R01HL092196, R01DK090908, P01HL068686 and P01HL074940), and by minigrants from the National Kidney Foundation of Maryland.

## Disclosures

Dr Jose, who is the Scientific Director of Hypogen, Inc, owns US Patent Number 6 660 474 for G protein–related kinase mutants in essential hypertension. The other authors report no conflicts.
